# Effect of estradiol with or without micronized progesterone on cholinergic-related cognitive performance in postmenopausal women

**DOI:** 10.3389/fnins.2024.1428675

**Published:** 2024-08-08

**Authors:** Alexander C. Conley, Jennifer N. Vega, Julia V. Johnson, Julie A. Dumas, Paul A. Newhouse

**Affiliations:** ^1^Center for Cognitive Medicine, Department of Psychiatry, Vanderbilt University Medical Center, Nashville, TN, United States; ^2^Department of Obstetrics, Gynecology and Reproductive Sciences, University of Vermont Larner College of Medicine, Burlington, VT, United States; ^3^Clinical Neuroscience Research Unit, Department of Psychiatry, University of Vermont Larner College of Medicine, Burlington, VT, United States; ^4^Geriatric Research, Education, and Clinical Center, Veterans Affairs Tennessee Valley Health System, Nashville, TN, United States

**Keywords:** estradiol, progesterone, cholinergic system, memory, attention, post-menopause

## Abstract

**Introduction:**

Women are at a higher risk of developing Alzheimer’s disease (AD), and the decline in estrogens post-menopause is thought of as a factor increasing this risk. Estradiol (E2) is important in supporting cholinergic neuronal integrity, and cholinergic functioning may be negatively impacted following the loss of E2 post-menopause. The use of exogenous E2 has been observed to enhance cholinergically mediated cognitive performance in healthy post-menopausal women, which indicates a potentially protective mechanism. However, E2 is often co-administered with progestin or progesterone to prevent endometrial proliferation. Progesterone/progestins have previously been shown to have a detrimental effect on E2-mediated biological and cognitive effects mediated by cholinergic systems in preclinical models, therefore the present study aimed to assess whether progesterone would modify the effect of E2 to influence cognition during cholinergic blockade.

**Methods:**

Twenty participants completed 3-months of oral E2 treatment with micronized progesterone (mPRO) or with placebo (PLC) in a repeated-measures within-subjects crossover design, in which they also completed five anticholinergic challenge days per hormone treatment condition. During the challenge participants were administered low or high doses of the nicotinic cholinergic antagonist mecamylamine, the muscarinic cholinergic antagonist scopolamine, or placebo. Following drug administration participants performed cognitive tests sensitive to cholinergic tone, assessing attention, episodic memory, and working memory.

**Results:**

Significant decrements were found on some tasks when participants were taking E2+mPRO compared to E2 alone. Specifically, under more challenging task conditions and larger anticholinergic doses, participants showed poorer performance on the Critical Flicker Fusion task and the Stroop test and responded more conservatively on the N-back working memory task. Other tasks showed no differences between treatments under cholinergic blockade.

**Discussion:**

The findings show that mPRO when taken in concert with E2, was detrimental to effortful cognitive performance, in the presence of cholinergic blockade. These results are important for assessing the impact of combined postmenopausal hormone treatment on cognitive performance that is dependent on cholinergic functioning after menopause.

## 1 Introduction

Women are at a higher risk of developing Alzheimer’s disease (AD) than men (Mazure and Swendsen, 2016; Koran et al., 2017; Mosconi et al., 2017; Ferretti et al., 2018; Laws et al., 2018; Oveisgharan et al., 2018). One of the proposed mechanisms that may influence this increased risk is menopause, which results in alterations of sex hormone levels that may affect brain function (Halbreich et al., 1995; Henderson, 2008; Henderson et al., 2016; Maki and Henderson, 2016; Conde et al., 2021). Complaints of cognitive decline are common during and following menopause in women (Weber and Mapstone, 2009) and may be related to the decline in circulating estradiol (Newhouse et al., 2013). Research studies suggest that estrogen levels influence memory, information processing speed, and executive functioning (Hogervorst et al., 2012), and may act in part through the cholinergic system to benefit cognition (Dumas et al., 2006, 2008, 2012; Gibbs, 2010). The loss of estrogens and particularly estradiol (E2) following menopause is thought to influence the activity of many neurotransmitter systems in the brain, however, the effects this depletion has on the cholinergic system may be particularly important for the future risk of AD (Newhouse and Dumas, 2015). This is because the cholinergic system is important for both attention and memory processes (Sarter et al., 2001; Parikh and Sarter, 2008; Hasselmo and Sarter, 2011; Ballinger et al., 2016), and the decline of cholinergic tone is directly linked to cognitive decline (Bartus et al., 1982; Dumas and Newhouse, 2011; Mesulam, 2012; Shen and Wu, 2015; Bohnen et al., 2018; Richter et al., 2018). Exogenous E2 has been shown to modulate cholinergic tone in both human (Bartholomeusz et al., 2008; Smith et al., 2011; Newhouse and Dumas, 2015) and animal models (Gibbs et al., 1994; Gibbs, 2000; Tinkler and Voytko, 2005; Gibbs, 2010; Mennenga et al., 2015).

Exogenous estrogen may provide a buffer against declining efficiency in cognitive processing resulting from the loss of sex hormones after menopause and subsequent effects on cholinergic system functioning (Gibbs, 1998). Thus, the use of post-menopausal hormone therapy (HT) using E2, or other estrogens or selective estrogen receptor modulators (SERMS) has been suggested as a potential neuroprotective strategy (Amtul et al., 2010; Azcoitia et al., 2011; Newhouse et al., 2013). There is evidence from epidemiological studies that show that HT can be effective at reducing the incidence of future dementia diagnoses (Zandi et al., 2002; Saleh et al., 2023). There is also some evidence that the timing of HT may be important in determining benefit, with better outcomes being seen in women who were prescribed HT in the first few years after menopause, compared to at least 5 years after menopause (Whitmer et al., 2011; Maki, 2013; Jamshed et al., 2014). However, results have not been unanimously positive, with other studies showing a lack of effect (Henderson et al., 2016), and in some cases is detrimental to cognitive performance (Maki et al., 2007). However, the explanation for variable HT effects on cognition may also be due to the other medications prescribed as part of HT, including progestins/progesterone.

When undergoing HT, all postmenopausal women with an intact uterus are co-prescribed progesterone, either a synthetic progestin or micronized progesterone, along with E2 to prevent endometrial hyperplasia. Similar to E2, progesterone/progestins impact a number of cortical processes. These hormones bind to progesterone receptors in the brain and are involved in the modulation of neurotransmitters such as dopamine, gamma-aminobutyric acid (GABA), glutamate and acetylcholine (Leavitt et al., 1987; Barth et al., 2015; Silvia et al., 2022). Progesterone/Progestins has been thought of as a potential neuroprotective agent like E2 for reducing the risk of future cognitive decline (Liu et al., 2010). However, progestins/progesterone prescribed as part of HT have varied effects on cognitive performance (Griksiene et al., 2022). The most common progestin that is prescribed as part of HT is medroxyprogesterone acetate (MPA), however, its impact on cognition in both animal models or in randomized controlled trials has been either negligible or detrimental to cognitive performance. In models examining the effects of different hormone treatments on ovariectomized animals, MPA has been shown to dampen or abolish beneficial effects seen with E2 alone, specifically in memory tasks like the Morris Water Maze or Radial Arm Maze (Bimonte-Nelson et al., 2006; Lowry et al., 2010; Braden et al., 2011). Moreover, when compared with other progestins, MPA impaired memory performance in ovariectomized rats to the greatest extent (Lowry et al., 2010; Braden et al., 2017). In humans, trials that assessed the impact of MPA have shown reduced verbal memory performance vs. placebo (Maki et al., 2009; Sherwin and Grigorova, 2011), however, others have shown improved visual memory vs. placebo (Resnick et al., 2006). In women who had undergone a hysterectomy, 24 weeks of E2 paired with MPA showed no difference in cognitive performance compared to E2 alone or placebo (Wolf et al., 2005). Micronized progesterone (mPRO) has been observed to improve working memory when paired with E2 compared to other HT regimens (Sherwin and Grigorova, 2011). These effects do not seem to persist when mPRO is given alone, as a small study of healthy postmenopausal women showed that 3 weeks administration of 300 mg mPRO alone did not improve either attention or verbal memory compared to placebo (Schüssler et al., 2008). Based on the results from these studies, there appears not to be a consistent effect of progesterone/progestins on cognitive performance in healthy postmenopausal women.

It can be challenging to directly examine the impact of exogenous sex hormones on cognitive performance in healthy postmenopausal women, as they may perform close to optimal levels thus enhancement may be difficult to detect. A potential solution to this difficulty is to assess the ability to compensate during a medication challenge that would otherwise impair performance. An effective challenge that has been used to examine cognitive performance in cognitively unimpaired women is the cholinergic challenge model (Drachman et al., 1980; Newhouse et al., 1988a,b; Snyder et al., 2005, 2014; Baakman et al., 2017). This pharmacological challenge model uses scopolamine (SCOP), a muscarinic antagonist, or mecamylamine (MECA), a nicotinic antagonist, to temporarily reduce cholinergic tone throughout the brain, which results in reduced cognitive performance (Newhouse et al., 1988a,b; Baakman et al., 2017). Cognitive performance under this anticholinergic challenge is then compared to performance under placebo, and the difference between these two challenge sessions shows the quantitative impact of the cholinergic blockade. When combined with hormone administration, this model allows assessment of the effects of hormones such as exogenously administered E2 on the impact of cholinergic antagonists on performance. Using this model, we have previously found that 3 months of E2 administration to cognitively normal postmenopausal women blunted the negative effects of SCOP and MECA on attention and memory tasks (Dumas et al., 2006, 2008). These results and others showed that E2 also modulated cortical activation in the brain under cholinergic blockade (Dumas et al., 2012) suggested that exogenous E2 was effective at compensating for temporary cholinergic dysfunction in cognitively normal postmenopausal women. However, it is not known whether the addition of progesterone/progestins alters the effects of E2 on the cognitive impact of cholinergic blockade in postmenopausal women.

The present study aimed to assess whether adding mPRO in combination with E2 treatment for 3 months would modify the previously observed effect of E2 to partially blunt the effect of the anticholinergic blockade on cognitive performance in cognitively normal postmenopausal women. To do this we examined the effectiveness of 3 months of E2 treatment plus mPRO versus 3 months of E2 plus placebo treatment (PLC) on cognitive performance under challenge by MECA or SCOP. This was accomplished using a cross-over design in which participants received both HT regimens, with a three-month placebo washout period in between. Based on our prior studies in humans and pre-clinical data, we hypothesized that the addition of mPRO to 3 months of E2 treatment compared to E2 treatment alone would result in a reduction of any beneficial effect of E2 on cognitive performance following acute muscarinic or nicotinic cholinergic blockade (i.e., E2 + PLC > E2 + mPRO). The impact of mPRO in combination with E2 on cognition was assessed using tests of attention, arousal, as well as episodic memory and working memory used in prior studies that were sensitive to the effects of cholinergic antagonists.

## 2 Methods

### 2.1 Participants and enrollment criteria

Participants recruited for this study were cognitively normal postmenopausal women over the age of 50. Participants were recruited through local newspaper advertisements, health newsletters published by the University of Vermont (UVM), and direct mail to randomly selected women over the age of 50 from a commercial mailing list. The current study protocol was approved by the UVM Institutional Review Board, and all participants gave written informed consent in accordance with the Declaration of Helsinki. Inclusion criteria included: (1) women over the age of 50, (2) without menses for at least *1 year*, and (3) have an FSH level greater than 30 mIU/ml, and (4) current non-smokers. Participants were physically examined by a gynecologic nurse-practitioner from the UVM Clinical Research Center for specific physical contraindications for E2 therapy (e.g., adnexal mass, large uterine fibroids, etc.) Participants were excluded for: (1) surgically induced menopause (bilateral oophorectomy), (2) current HT or HT within the last year, (3) current Axis I or II psychiatric or cognitive disorders (see screening below), (4) specific physical contraindications for E2 therapy (e.g., adnexal mass, large uterine fibroids, etc). Exclusion criteria are included in the Supplementary material.

As part of the screening procedures, participants completed several clinical and cognitive measures. Global cognitive performance was assessed by the Mini-Mental State Exam (MMSE) (Folstein et al., 1975). Cognitive performance was assessed utilizing the Mattis Dementia Rating Scale (DRS-2) (Mattis, 1988). Depressive symptoms were assessed by the Beck Depression Inventory (BDI) (Beck et al., 1961), and a partial Structured Clinical Interview for DSM-IV (SCID-IV) (First et al., 1997). Menopausal symptoms were collected by the Menopause Symptom Checklist (MSC) (Newhouse et al., 2010). Participants’ functioning was rated by examiners on the Brief Cognitive Rating Scale (BCRS) (Reisberg and Ferris, 1988) and the Global Deterioration Scale (GDS) (Reisberg et al., 1982).

### 2.2 Study design and procedure

**Figure 1:**
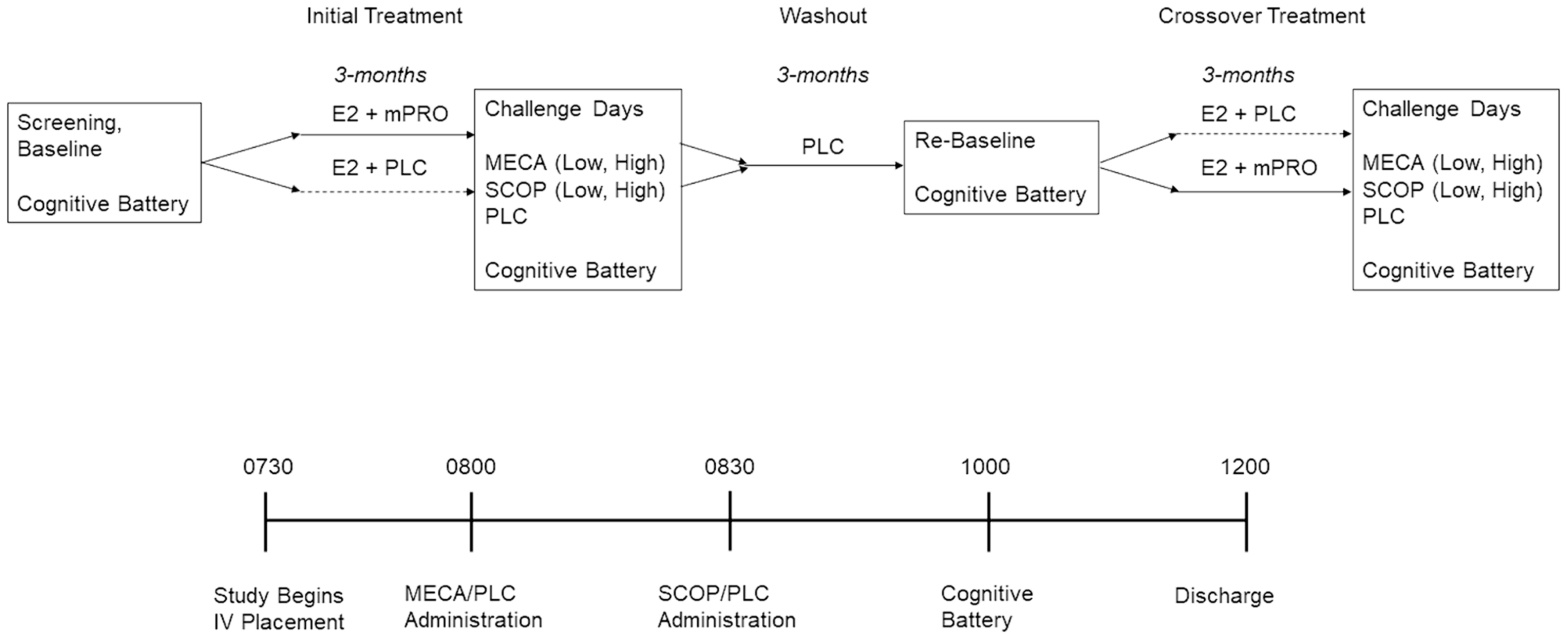
Experimental design of the treatment period and challenge days. E2, estradiol; MECA, mecamylamine; mPRO, micronized progesterone treatment; PLC, placebo treatment or challenge day; SCOP, scopolamine.

The study was a double-blinded randomized hormone treatment crossover study. Figure 1 outlines the overall procedure of the study, as well as the procedure during anticholinergic challenge days. After the initial screening session, participants completed baseline cognitive assessments. The cognitive battery used at baseline was the same used on each study day and consisted of tasks designed to measure arousal, attention, and memory (see below for description). After baseline participants were randomly assigned to receive oral 17β-estradiol (E2) and 200 mg micronized progesterone (mPRO) or placebo (PLC). The 3-month treatment period consisted of receiving 1 mg oral E2 for 1 month and then 2 mg oral E2 for the next 2 months. Following this treatment period, participants completed the five anticholinergic challenge days. Following the completion of the challenge days, participants completed 3 months of treatment washout, where they received two pills that were both placebo. After the washout period, the participants completed a cognitive battery to re-baseline their performance before beginning the 3-month crossover treatment period in which they received the other treatment regimen (E2 + mPRO or E2 + PLC). Once again, at the end of the 3-month treatment period, they completed the five anticholinergic challenge days. At the end of the second treatment phase and after all challenges were completed, participants were administered 10 mg of medroxyprogesterone acetate (MPA) per day for 12 days to produce endometrial shedding. To verify compliance, pill counts were performed at the end of each 3-month treatment phase visit. FSH and estradiol levels were measured before each challenge sequence.

The five anticholinergic days were: 10 mg oral mecamylamine (Low-MECA) + intravenous (IV) placebo; 20 mg oral mecamylamine (High-MECA) + IV placebo; 2.5 μg/kg IV scopolamine (Low-SCOP) + oral placebo; 5 μg/kg IV scopolamine (High-SCOP) + oral placebo; or an oral and IV placebo (PLC). The order of challenge days was randomized across participants, and investigators were blinded to the drugs being administered. The procedure for the testing days was as follows.

Participants were admitted to the Clinical Research Center at UVM in the morning at 0730. At this time, an IV was started with saline continuously delivered for the next 7 h to reduce the peripheral effects of the antagonists. Participants received an oral dose of mecamylamine or placebo at 0800 and at 0830 an intravenous dose of scopolamine or placebo depending on treatment assignment as indicated above. Cognitive testing began at 1000, at the estimated peak drug effect time (based on prior work) for the cholinergic antagonists taken 1.5 h for SCOP or 2 h for MECA prior. The order of the cognitive tasks was counterbalanced across the challenge days. Participants were discharged at approximately 1200, or 5 h following the beginning of the saline administration. All participants were fasting since the night before on the day of testing. The five challenge days were completed across a three-week period in which all challenge days were separated by at least 48 h to ensure challenge drug washout before the next challenge day (Newhouse et al., 1988a,b; Putcha et al., 1989; Newhouse et al., 1992, 1994; Ebert et al., 2001; Young et al., 2001; Baakman et al., 2017).

### 2.3 Cognitive battery

The critical flicker fusion task or CFF (Kupke and Lewis, 1989) is a test of attention/vigilance using the frequency of a flickering LED. In an ascending trial, the participant pressed a button indicating when the frequency of flashing lights (beginning at 12 Hz and increasing to 50 Hz), had increased to the point that the lights appear to be no longer flashing but rather appear continuously on (“fused”). In a descending trial, beginning at 50 Hz, the participant pressed a button when the frequency of apparently fused lights was decreased such that lights began to appear to be flashing. The participant needed to respond before the frequency hits the upper or lower limit in each trial. The outcome variable for CFF is frequency (Hz) for ascending and descending trials.

The choice reaction time or CRT (Hindmarch, 1984) task is a measure of attention and psychomotor speed. The CRT task is a reaction time task in which participants were asked to keep their index finger on a “home” key next to a liquid crystal diode (LCD) until one of 6 LCDs arrayed in a semicircle, approximately 25 cm from the “home” key, was lit on the response box. When one of the 6 LCDs arrayed in a semicircle illuminates, the participant was asked to lift her index finger and press the corresponding button next to the illuminated LCD, then return her finger to the “home” LCD button. This pattern continues for 50 trials. Outcome variables on the CRT include the mean and median total reaction time, which can be separated into recognition time (time from stimulus onset to initiation of movement) and motor time (time from initiation of movement to stimulus termination). Lower scores indicate better performance.

A visually presented N-back sequential letter task assessed working memory performance (Jonides et al., 1997; Saykin et al., 2004). Four conditions were presented: 0-back, 1-back, 2-back, and 3-back. The 0-, 1-, 2-, and 3-back conditions were performed in two blocks of 27 trials each for a total of 216 trials. The main outcome variables of the n-back task were the sensitivity (*d’*), calculated as: standardized (Hits) – standardized (False Alarms); or the response bias (C) calculated as: – [standardized (Hits) + standardized (False Alarms)]/2. The sensitivity measures the ability of the participant to discriminate between match or mismatch (Stanislaw and Todorov, 1999). The response bias measure relates to the likelihood of participants responding to a match vs. mismatch (Snodgrass and Corwin, 1988; Stanislaw and Todorov, 1999). Positive response biases reflect a more conservative criterion, with a greater likelihood of responding mismatch, which reduces noise but also signal. Negative response biases reflect a more liberal criterion, with a greater likelihood of responding match, which increases the likelihood of signal but also noise. Neutral response biases of 0 reflect no greater likelihood of reducing signal or increasing noise in response style. These two variables were assessed for each of the four conditions.

A 2-alternative forced-choice test was used to test recognition memory (Hicks and Marsh, 1998). The participants were first shown a series of words and asked to count the number of vowels in each of the words. Next, participants were shown a list of words, some of which were shown previously, and some that were new words. Participants were asked to indicate whether the word on the screen was a new word they had not seen in the prior list or an old word that had just appeared on the list. Measures of sensitivity (*d’*) and response bias (C) were calculated for analysis.

A color word Stroop test was used to test selective attention (Lansbergen and Kenemans, 2008). Neutral, congruent, and incongruent stimuli were presented in different blocks across each challenge day. Key outcome variables that were assessed were the accuracy and mean response time for correct responses for both congruent and incongruent stimuli.

### 2.4 Statistical analyses

Analyses for each cognitive task used the outcome variables specified above. The analyses on the outcome variables were performed in the following manner. To examine any changes across hormone treatment without cholinergic blockade, a comparison was performed between hormone treatments on the outcome variables on each placebo challenge day. To assess the difference in training that may have occurred across the study, we also examined the performance of participants on cognitive tasks between their initial baseline testing day prior to their first hormone treatment, and the re-baselining that occurred before the second set hormone treatment. Following these comparisons, the effect of the cholinergic blockade on performance under each hormone treatment was compared. To do this difference scores were calculated for each of the outcome variables as the anticholinergic challenge performance minus placebo challenge performance. To examine the impact of differing doses of the blockade of nicotinic or muscarinic receptors on cognitive performance MECA and SCOP challenge days were analyzed separately.

The analysis of the placebo challenge day performance used a generalized linear model with one within-subjects variable of hormone treatment (E2 + mPRO vs. E2 + PLC) for all tasks except the Stroop task, in which the performance was assessed by a generalized linear model with two within-subjects variables of hormone treatment and trial type (congruent vs. incongruent). When assessing the anticholinergic performance, the analyses of all tasks except the Stroop task were assessed by generalized linear models with two within-subjects variables of hormone treatment and challenge dose (low dose vs. high dose). For the Stroop task, the outcome variables were assessed by a generalized linear model with three within-subjects variables, hormone treatment, challenge, and trial type. Age was included as a covariate in these models. Analyses were performed using the R statistical software version 4.3.1. Due to the challenging nature of this study with lengthy treatment phases and 10 pharmacological challenge days, there were some participants who did not complete all challenge days. Statistical analyses were completed using the lme4 package (Bates et al., 2015) that handles data missing at random and all generalized linear models included a random intercept. Analyses tables used the clubsandwich (Pustejovsky, 2023) and sjPlot for visualization (Lüdecke, 2023), and effect sizes were calculated using the RESI package (Jones et al., 2023). All analyses report estimates and 95% confidence intervals (CI) for generalized linear models.

## 3 Results

Demographic information for participants is outlined in Table 1. Twenty cognitively unimpaired postmenopausal women participated in the study (mean age: 63.8 ± 9.02 years). The participants had 15.5 ± 2.3 years of education and an average IQ of 125 ± 8.52. The participants were on average 13.8 ± 8.6 years post-menopause, and 16 women were in the late stage of menopause according to the Stages of Reproductive Aging Workshop’s + 10 staging system (Harlow et al., 2012). Two women had prior hysterectomies, but not a bilateral oophorectomy. Of the 20 participants, 14 had previously used HT, and the mean length of prior hormone use was 7.43 ± 4.87 years. No participants took HT within the last year prior to their participation.

**Table 1:** Demographic information.

Measure	Mean (SD)	Range
Age (years)	63.8 (9.02)	51–82
Education (years)^	15.5 (2.3)	12–18
Years since menopause	13.8 (8.61)	1–30
Prior HT (yes/no)	14/6	
Length of HT (years)	7.43 (4.87)	1–15
Natural menopause (yes/no)	18/2	
MMSE	29.2 (0.77)	28–30
IQ	125 (8.52)	110–144
DRS raw score	141 (1.73)	138–144
BCRS	8.5 (0.61)	8–10
GDS	1.45 (0.51)	1–2
BDI	2.95 (3.44)	0–13
MSC	17.6 (11.2)	0–35

Numbers represent mean scores with standard deviations in parentheses unless specified. MMSE, Mini-Mental State Exam; DRS, Mattis Dementia Rating Scale-2; BCRS, Brief Cognitive Rating Scale; GDS, Global Deterioration Scale; BDI, Beck Depression Inventory; MSC, Menopause Symptom Checklist. ^*n* = 19.

The comparison between pre-treatment baseline cognitive testing and the re-baselining between the first and second treatment revealed some small but statistically significant differences in cognitive performance (see Supplementary Data for detailed results). Performance on the CFF ascending trial increased between the initial baseline performance and the post-phase 1 treatment baseline by approximately 2 Hz (Estimate: 1.75, 95% CI [0.55, 2.96], *p* = 0.005). Mean response time on the Stroop task also improved between the initial baseline and the re-baselining session after the first treatment phase (Estimate 63.9, 95% CI [20.2, 108], *p* = 0.007). These differences, while significant were small in magnitude and were adjusted for in the challenge day analyses by using change scores from placebo challenge day performance.

### 3.1 Placebo challenge day performance

Results of the analyses for the placebo challenge day performance are displayed in Table 2. There were no significant differences between hormone treatments during the placebo challenge performance on any of the tasks except for the N-back and the Stroop task. On the N-back task, there was a significant effect of hormone treatment found for response bias on the 3-back task, with participants responding more conservatively while taking E2 + PLC compared to when they were taking E2 + mPRO (Estimate: 0.19, 95% CI [0, 0.38], *p* = 0.048). On the Stroop task, there was a significant difference in accuracy across the hormone treatments with slightly decreased accuracy for the E2 + mPRO treatment phase (E2 + mPRO: 96.9% ± 1.2, E2 + PLC: 96.2% ± 2.3; Estimate: 1, 95% CI [0, 2], *p* = 0.038).

**Table 2:** Placebo challenge day results across the hormone treatment phases.

Task	Variable	E2 + PLC	E2 + mPRO
CFF	Median ascending (Hz)	30.2 (4.6)	29.5 (3.5)
	Median descending	30.5 (4.4)	31.2 (5.4)
CRT	Median total RT (ms)	887 (143)	891 (161)
	Median recognition RT	445 (53.9)	445 (59.8)
	Median motor RT	430 (111)	436 (121)
N-back	0-back sensitivity (d’)	2.53 (3.04)	2.78 (2.73)
	1-back sensitivity	1.95 (2.69)	2.32 (2.6)
	2-back sensitivity	1.49 (2.19)	1.64 (2.13)
	3-back sensitivity	1.04 (1.63)	1.37 (1.6)
	0-back bias (C)	0.164 (0.2)	0.18 (0.13)
	1-back bias	0.01 (0.4)	0.08 (0.13)
	2-back bias	0.23 (0.3)	0.26 (0.31)
	3-back bias^a^	0.62 (0.4)	0.42 (0.42)
Recognition memory	Sensitivity (d’)	2.61 (0.75)	2.49 (0.8)
	Bias (C)	0.21 (0.59)	0.18 (0.56)
Stroop task	Congruent accuracy (% correct)^a^	95.6 (0.6)	94.6 (2.3)
	Incongruent accuracy	98.3 (2.0)	97.8 (2.3)
	Congruent RT (ms)	719 (147)	707 (137)
	Incongruent RT	989 (281)	1,009 (321)

E2, estradiol; PLC, placebo treatment; mPRO, micronized progesterone treatment; CFF, critical flicker fusion task; CRT, choice reaction time task. Numbers represent mean scores on the placebo challenge day with standard deviations in parentheses. ^a^Significant difference between hormone treatment phases (*p* < 0.05).

### 3.2 Anticholinergic challenge day performance

#### 3.2.1 Tests of attention and arousal

**Figure 2:**
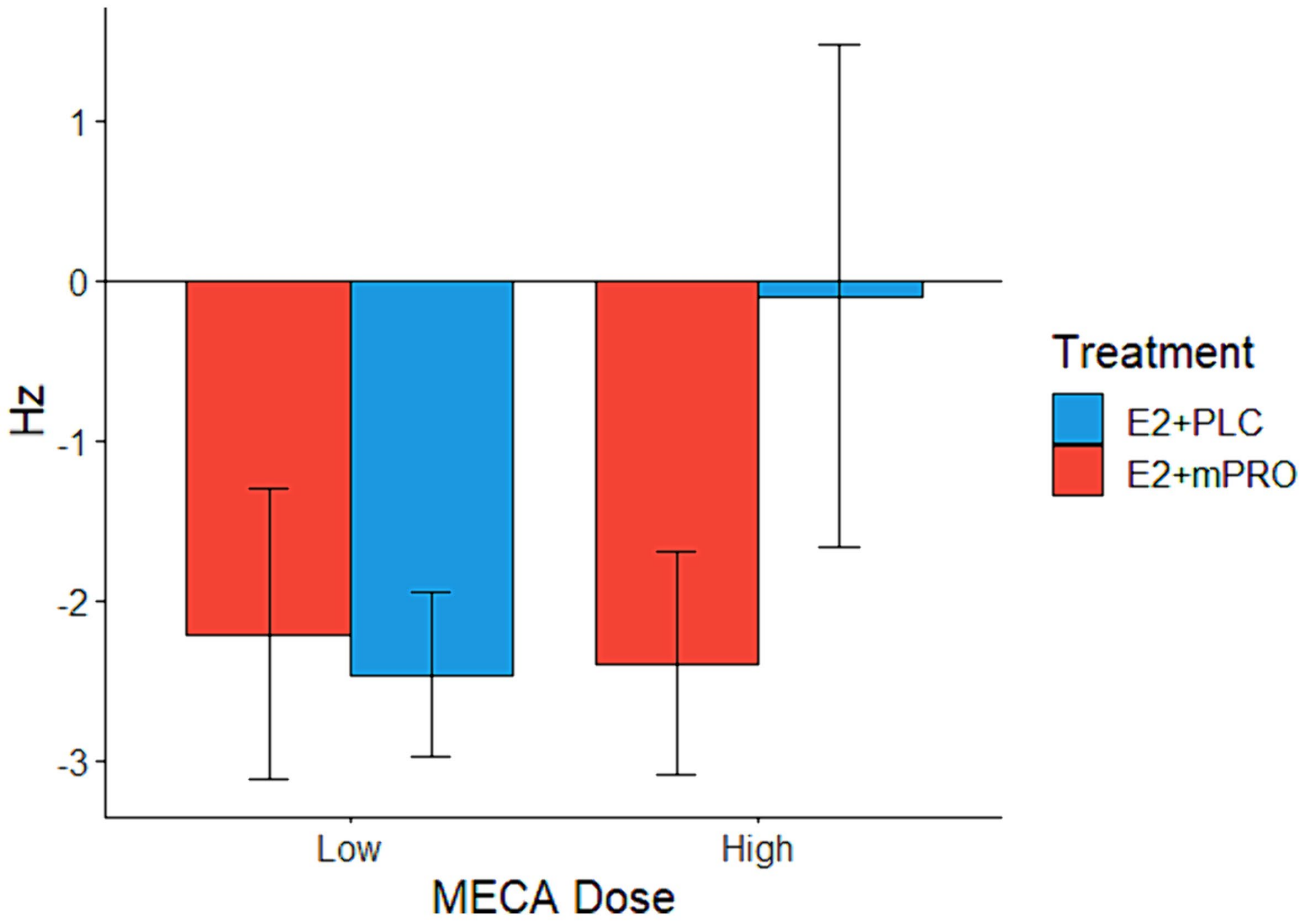
Median descending score on the Critical Flicker Fusion task across both hormone treatments on the MECA challenge days. Performance indicates the difference in the descending score between the active challenge day and the placebo challenge day (Active – Placebo). A significant interaction was found between hormone treatments and MECA dose (*p* = 0.025), where participants taking E2 + PLC responded similarly on both the placebo and the high MECA challenge days to descending trials, in comparison to the low MECA challenge day, and when they were taking E2 + mPRO.

Results of the attention task performance on the anticholinergic challenge days are displayed in Table 3. Performance across all treatment phases and challenge doses declined compared to performance under the placebo challenge day. The analysis of the median ascending score of the CFF showed worse performance for both MECA and SCOP conditions compared to placebo, however, this decrease in performance did not differ across the treatment phase or challenge drug doses (all *p* > 0.3). The analysis of the median descending score showed a significant interaction between hormone treatment and CD dose on the MECA challenge days (Figure 2; Estimate = 3.08, 95% CI [0.39, 5.76], *p* = 0.025). This effect was driven by participants performing better on the high MECA challenge day while taking E2 + PLC compared to the low MECA challenge day, or either MECA challenge day when taking E2 + mPRO. There was also a significant main effect of MECA dose (Estimate = 2.69, 95% CI [0, 5.38], *p* = 0.05). There were no significant effects observed on the SCOP challenge day on either the effects of hormone treatment or challenge day dose.

**Table 3:** The performance difference on the attention and arousal tasks for both hormone treatments on the anticholinergic challenge days.

Task	Variable	E2 + PLC				E2 + mPRO			
Low-MECA		High-MECA	Low-SCOP	High-SCOP	Low-MECA	High-MECA	Low-SCOP	High-SCOP
CFF	Median ascending (Hz)	−1.49 (2.96)	−1.37 (2.79)	−1.9 (2.78)	−2.74 (3.1)	−0.88 (1.36)	−0.92 (3.62)	−1.2 (2.96)	−2.98 (3.86)
	Median descending^a^	−2.46 (2.16)	−0.09 (6.7)	−2.11 (7.12)	−1.35 (8.03)	−2.21 (3.75)	−2.39 (3.13)	−2.98 (3.86)	−2.16 (3.98)
CRT	Median total RT (ms)	52.4 (98.9)	46.4 (111)	72.4 (93.2)	149 (134)	11.9 (84.8)	24 (81.6)	79.5 (112)	150 (164)
	Median recognition RT	11.2 (44.2)	1.64 (49.9)	14.7 (41.6)	50.3 (55.1)	−4.47 (39.9)	2.42 (41.6)	24.4 (59.2)	58.1 (60.3)
	Median motor RT	41.9 (65.5)	42.8 (76.7)	49.1 (81.8)	90.3 (106)	8.72 (60.7)	20.6 (52.7)	50 (58.9)	72.4 (122)
Stroop task	Congruent accuracy (% correct)	−0.44 (1.0)	−0.35 (1.32)	−2.06 (3.17)	−2.06 (2.84)	0.39 (2.35)	0.4 (1.76)	−0.35 (2.68)	−2.21 (4.5)
	Incongruent accuracy	−1 (3.93)	−0.77 (3.0)	−3.78 (5.25)	−8.53 (9.0)	−1 (3.0)	−0.79 (1.72)	−5.11 (5.17)	−12.2 (13.5)
	Congruent RT (ms)^b^	37.2 (79.2)	25.6 (91.8)	82 (142)	83.9 (164)	5.59 (85.3)	12.6 (76.9)	41.5 (95.4)	225 (346)
	Incongruent RT	84.9 (174)	61.1 (157)	68.7 (146)	204 (336)	−46 (114)	26.1 (136)	50.3 (156)	280 (494)

E2, estradiol; MECA, mecamylamine; mPRO, micronized progesterone treatment; PLC, placebo treatment; SCOP, scopolamine; CFF, Critical flicker fusion task; CRT, choice reaction time task. Numbers represent difference scores of challenge day (active drug – placebo), with standard deviations in parentheses. ^a^Significant interaction between hormone treatment and mecamylamine dose (*p* < 0.05). ^b^Significant difference between hormone treatment phases during scopolamine challenge days (*p* < 0.05).

Analysis of the CRT on the MECA challenge days showed no significant main effects or interactions for hormone treatment or challenge dose across the total, recognition, or motor time scores. On the SCOP challenge days, there was a significant effect of challenge dose observed across all the time measures (see Table 3). For all scores, participants responded slower following the High-SCOP compared to Low-SCOP (total time: Estimate = 75.52 ms, 95% CI [32.43, 118.6], *p* = 0.001; recognition time: Estimate = 34.62 ms, 95% CI [16.33, 52.91], *p* < 0.001; motor time: Estimate = 41.31 ms, 95% CI [4.82, 77.8], *p* = 0.027). There was no difference between the hormone treatments, nor was there an interaction between hormone treatment and SCOP dose.

Results of the Stroop task on the MECA challenge days did not show any effect of hormone treatment, MECA dose, or trial type on either the accuracy or response times of the participants. Analysis of the accuracy of the Stroop task for SCOP challenge days revealed a significant interaction between SCOP dose and trial type, with worse accuracy on incongruent trials following the higher dose of SCOP compared to the lower dose of SCOP (Estimate = 5, 95% CI [2, 8], *p* < 0.001). There was also a main effect of trial type, with lower accuracy for incongruent compared to congruent trials (see Table 3; Congruent: −1.99 ± 3.46 vs. Incongruent: −8.29 ± 9.07% correct; Estimate = 6, 95% CI [3, 10], *p* < 0.001). There was no effect of hormone or interaction between hormone and SCOP dose or trial type.

**Figure 3:**
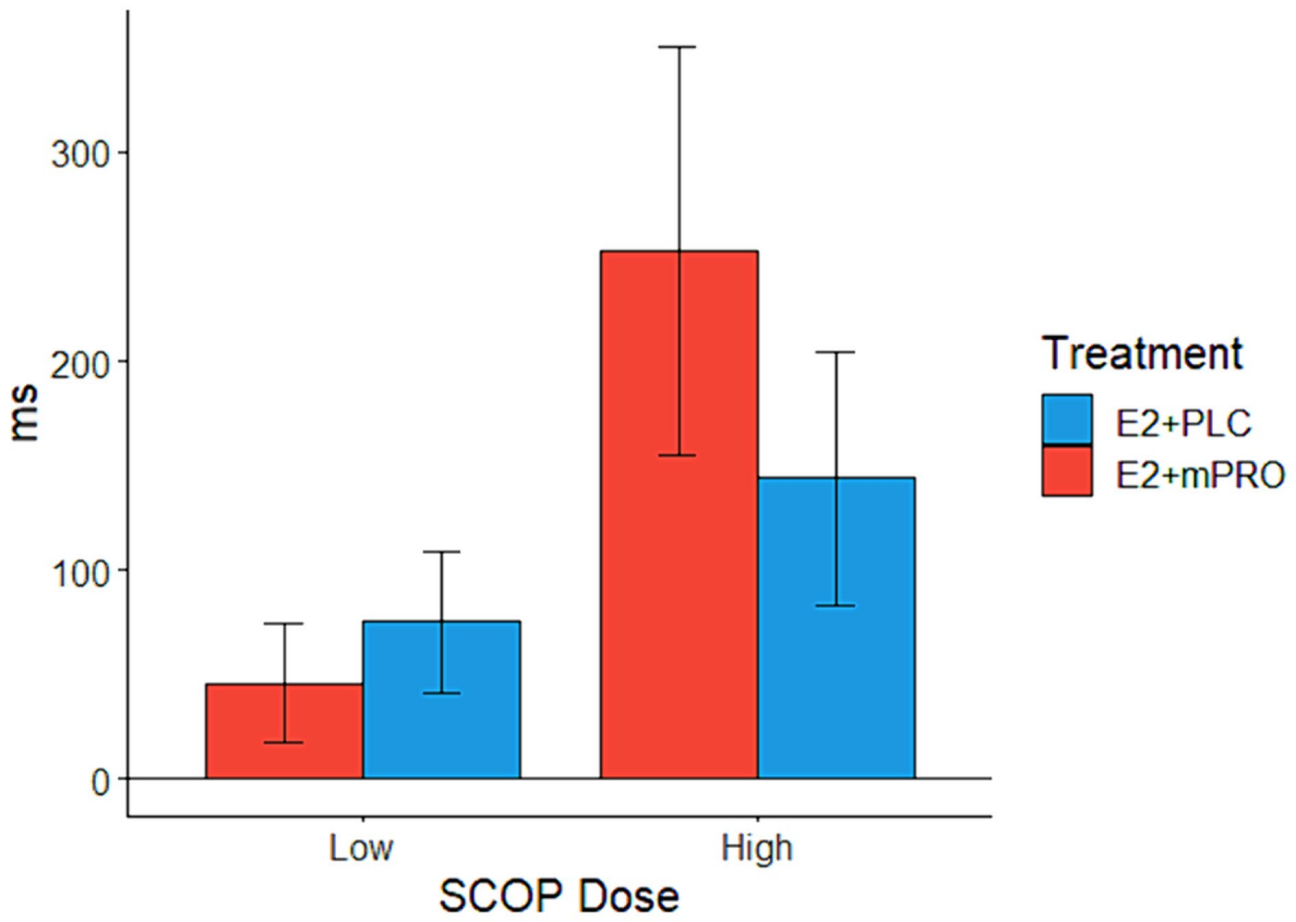
Mean response times on the Stroop task across both hormone treatments on SCOP challenge days. Performance indicates the difference in the mean response times between the active challenge day and the placebo challenge day (Active – Placebo). A significant interaction was observed between hormone treatments and SCOP dose (*p* = 0.011), which was driven by when participants taking E2 + mPRO responded slower than when they were taking E2 + PLC, particularly for the higher SCOP challenge days.

Analysis of the response times on the Stroop task for the SCOP challenge days revealed an interaction between hormone treatment and SCOP dose (Figure 3; Estimate = 183.67 ms, 95% CI [43.01, 324.33], *p* = 0.011). On low-dose SCOP challenge days, participants responded slightly slower when they were taking E2 + PLC compared to when they were taking E2 + mPRO (45.9 ± 125.7 ms vs. 75.4 ± 144 ms). On high-dose SCOP challenge days, participants responded much slower when they were taking E2 + mPRO compared to when they were taking E2 + PLC (144 ± 250 ms vs. 252.5 ± 420 ms). There was also a significant main effect of the hormone treatment on response times, with participants responding slower when taking E2 + mPRO compared to when taking E2 + PLC (Estimate = 148.09 ms, 95% CI [40.28, 255.9], *p* = 0.007). In addition to the effects of hormone treatment, there was a significant interaction observed between trial type and SCOP dose, with response times on incongruent trials being more affected by SCOP dose than congruent trials (Estimate = 133.78 ms, 95% CI [17.73, 249.83], *p* = 0.024). Similarly, a significant main effect of trial type was observed with participants responding faster for congruent vs. incongruent trials (Estimate =120.49 ms, 95% CI [18.8, 222.18], *p* = 0.021).

#### 3.2.2 Tests of memory

**Figure 4:**
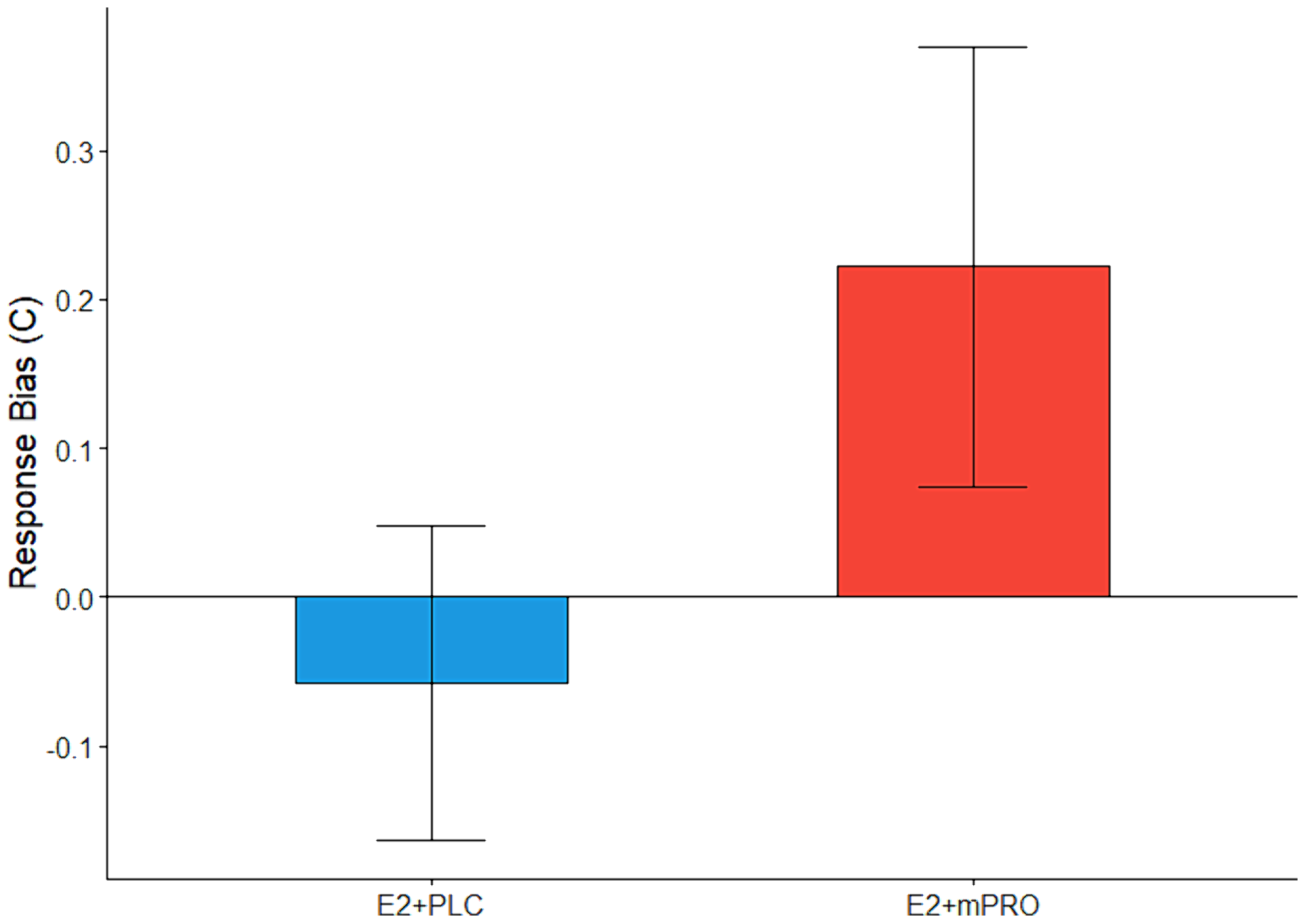
The difference across treatment phases on the response bias on 3-back during the SCOP challenge days. Performance indicates the difference in response biases between the active challenge day and the placebo challenge day (Active – Placebo). A significant main effect of hormone treatment was observed (*p* = 0.017), with participants responding more conservatively when taking E2 + mPRO on the SCOP challenge days compared to when they were taking E2 + PLC.

Results of the memory task performance of participants during the anticholinergic challenge days are displayed in Table 4. Analysis of the N-back task showed that across both MECA and SCOP challenge days, there were no significant differences observed for sensitivity (*d’*), either in relation to hormone treatment or challenge day dose. There were also no significant differences observed for response bias on MECA challenge days. For the SCOP challenge days, there was a significant difference in response bias for the 3-back task between hormone treatments (Figure 4; Estimate = 0.34, 95% CI [0.06, 0.62], *p* = 0.017). Participants elicited a larger response bias on SCOP challenge days compared to placebo challenge days when they were taking E2 + mPRO compared to when they were taking E2 + PLC. Analysis of the performance on the recognition memory task showed no significant differences for either MECA or SCOP challenge days across hormone treatment, challenge dose, or the interaction between hormone treatment and the challenge dose.

**Table 4:** The performance difference on episodic and working memory tasks for both hormone treatments on the anticholinergic challenge days.

Task	Variable	E2 + PLC				E2 + mPRO			
Low-MECA		High-MECA	Low-SCOP	High-SCOP	Low-MECA	High-MECA	Low-SCOP	High-SCOP
N-back	0-back sensitivity (d’)	0.32 (4.37)	0.88 (3.9)	0.51 (3.9)	−0.29 (3.4)	0.52 (3.5)	0.99 (2.5)	0.68 (3.1)	0.045 (3.45)
	1-back sensitivity	0.64 (3.84)	1.15 (3.22)	0.36 (3.35)	0.46 (3.1)	0.61 (3.18)	0.95 (2.6)	0.65 (2.93)	−0.29 (3.0)
	2-back sensitivity	0.57 (2.84)	0.98 (2.55)	0.37 (3.0)	0.11 (3.0)	0.75 (2.3)	0.7 (2.0)	0.61 (2.43)	−0.17 (2.91)
	3-back sensitivity	0.34 (2.34)	0.64 (0.21)	0.39 (2.3)	0.18 (2.1)	0.28 (1.55)	0.48 (1.42)	0.09 (1.73)	−0.32 (2.1)
	0-back bias (C)	0.03 (0.33)	0.06 (0.29)	0.17 (0.27)	0.2 (0.43)	0.02 (0.2)	0.12 (0.18)	0.12 (0.21)	0.02 (0.37)
	1-back bias	0.1 (0.38)	0.17 (0.45)	0.27 (0.54)	0.33 (0.48)	0.07 (0.24)	0.1 (0.29)	0.13 (0.24)	0.31 (0.35)
	2-back bias	−0.07 (0.39)	−0.03 (0.58)	0.05 (0.5)	0.18 (0.37)	0.04 (0.29)	0.04 (0.38)	0.1 (0.56)	0.1 (0.45)
	3-back bias^b^	−0.08 (0.28)	0.03 (0.47)	0.04 (0.43)	−0.07 (0.44)	0.18 (0.34)	0.21 (0.32)	0.16 (0.45)	0.28 (0.39)
Recognition memory	Sensitivity (d’)	−0.13 (0.62)	−0.14 (0.87)	−0.65 (0.8)	−1.11 (1.1)	−0.04 (0.7)	−0.15 (0.72)	−0.41 (0.82)	−0.87 (0.87)
	Bias (C)	−0.03 (0.43)	−0.004 (0.43)	−0.17 (0.48)	−0.13 (0.4)	0.11 (0.37)	−0.07 (0.31)	−0.1 (0.28)	−0.08 (0.4)

E2, estradiol; MECA, mecamylamine; mPRO, micronized progesterone treatment; PLC, placebo treatment; SCOP, scopolamine. Numbers represent different scores of challenge day (active drug – placebo), with standard deviations in parentheses. ^b^Significant difference between hormone treatment phases during scopolamine challenge day (*p* < 0.05).

## 4 Discussion

We investigated whether adding mPRO to 3-months of treatment with E2 would reduce the effect of E2 on cholinergic blockade on cognitive performance in postmenopausal women compared to 3 months of E2 plus placebo. The results across most measures of attention and memory showed minimal difference in performance between the two hormone treatment conditions when they were under cholinergic blockade. For comparisons where there were significant differences between the hormone treatments, the direction of the differences were consistent with our predictions, showing worse performance when participants were taking E2 + mPRO compared to when they were taking E2 + PLC.

The present study expands on our prior work as it explores the impact of progesterone on the ability of exogenous E2 to mediate anticholinergic blockade of cognitive performance in postmenopausal women. The results of the present study showed that when combined with E2 treatment, mPRO was detrimental to the performance of participants on the CFF, Stroop and N-back tasks. On the tasks of attention, the addition of mPRO negatively affected the performance of the participants, particularly when under higher levels of cholinergic blockade. On the CFF, participants’ performance during the high MECA challenge days was similar to the placebo challenge day when they were taking E2 + PLC, but there was a clear decrement in perception scores when participants were administered E2 + mPRO. On the Stroop task, there was a slight inverse relationship between SCOP dose and performance on the different hormone treatments. On the low SCOP challenge days, the participants were slightly faster when taking E2 + mPRO (29.5 ms), however on high SCOP challenge days they were much faster when taking E2 + PLC (108.5 ms). While there was no worsening of N-back accuracy when participants were taking E2 + mPRO, there was a clear effect of progesterone that altered response bias during the most difficult 3-back block after SCOP challenge. After placebo challenge, participants responded more conservatively when taking E2 + PLC, and more liberally when taking E2 + mPRO. The effect of SCOP, when participants were taking E2 + mPRO, was to shift the response biases to be more conservative, whereas there was no major effect of SCOP on participants’ responses while taking E2 + PLC. This shift in response bias with SCOP is consistent with previous research (Mintzer and Griffiths, 2001, 2003); however, the fact that the shift only occurred while participants were taking E2 + mPRO indicates that the potential mitigation of cholinergic antagonism by E2 was diminished by the addition of the progestin.

The lack of a beneficial effect of combining E2 with mPRO on cholinergically-mediated cognitive performance is consistent with several previous studies examining the impact of HT on cognitive performance in postmenopausal women (Wegesin and Stern, 2007; Maki et al., 2009; Sherwin and Grigorova, 2011; Henderson, 2018). The comparison between healthy postmenopausal women on E2 alone compared to those taking E2 combined with mPRO have shown that either no impact (Wolf et al., 2005; Schüssler et al., 2008), or detrimental effects on the performance of executive functions and recognition memory (Wegesin and Stern, 2007). Participants in the present study performed similarly on cognitive tasks while taking E2 + PLC and E2 + mPRO during the placebo challenge days, apart from differences in Stroop task accuracy and 3-back response bias. Under anticholinergic blockade, cognitive performance was similar on a number of tasks, with both hormone treatments failing to consistently mitigate the impact of either MECA or SCOP. However when there were differences between the hormone treatments, the performance of participants favored PLC over mPRO. The results indicated that the addition of mPRO to E2 leads to greater interference in some cognitive processes under cholinergic blockade. Additionally, when looking at these significant differences between the hormone treatments, they were most clear during the more difficult tasks in the cognitive battery. Both the Stroop test and the 3-back task were more difficult compared to other tasks in the cognitive battery. This finding indicated that mPRO impacted the ability of participants to compensate or mitigate for the cholinergic blockade when cognitive processes were under the most effortful conditions, compared to when they performed less challenging tasks. The results of the present study also indicate a difference in the effects of the different cholinergic antagonists in their interactions with the HT regimens. The HT effects for the more difficult tasks were observed during SCOP compared to MECA challenge days. In contrast, only the vigilance effect was seen during the MECA challenge days. These findings show that participants taking E2 + mPRO were less able to mitigate muscarinic blockade by SCOP compared to nicotinic blockade by MECA.

This study has a number of implications for women’s cognitive health and aging, as well as their risk for AD. This research builds upon previous literature showing the relationship of HT to cognitive performance in postmenopausal women. As discussed earlier, the blockade of cholinergic receptors creates a temporary “lesion” model, in which we can measure the impact on the ability to compensate for this blockade. As E2 regulates the cholinergic system, if the HT regimens to mitigate the effects of the blockade, they would be supportive of a potential neuroprotective effect. However, in the present study, neither hormone regimen was able to consistently mitigate the effects of the cholinergic blockade, and moreover, the E2 + mPRO regimen was more detrimental than the E2 + PLC regimen during more challenging tasks. These results are important as the ability to compensate for cholinergic deterioration may be important in reducing the risk of future cognitive decline, including the development of AD in older women. As cholinergic deterioration is related to cognitive impairment (Bartus et al., 1982; Fernández-Cabello et al., 2020), identifying what exogenous medications may alleviate, and those that exacerbate cholinergic deterioration is significant. For women who possess AD-risk factors including genetic predisposition, comorbidities, or biomarker burden, the impact that specific HT regimens may have on cholinergic functioning may be important for assessing risk for cognitive decline.

These results also have implications for the use of progestins in HT. Much of the previous research has focused on the detrimental impact of MPA on cognitive performance, either when administered alone or in conjunction with exogenous E2 (Resnick et al., 2006). Comparatively, mPRO has been shown to be less detrimental to the cognitive performance of women (Sherwin and Grigorova, 2011). The results of the present study reflect this relationship somewhat, as there were generally no differences in performance between the HTs on the placebo challenge days. However, during more effortful conditions, like during cholinergic blockade or with more challenging task conditions, mPRO was detrimental to performance. A potential mechanism that may be driving this effect is the interactions between E2 and progesterone in the brain. Previous research, mainly in animal models, has shown that progesterone administration may alter the effects of E2 when given together (Baudry et al., 2013). Progesterone alters the ability of E2 to regulate either the metabolism of brain mitochondria (Irwin et al., 2008), or the increase in neurotrophins (Bimonte-Nelson et al., 2004). In addition, the addition of progesterone was found to reduce E2-driven benefits in spatial memory in ovariectomized rats (Bimonte-Nelson et al., 2006). Another potential mechanism could be through the upregulation of inhibitory γ-Aminobutyric acid (GABA) receptors. In animal models, exogenous progesterone has been shown to boost the activity of GABA_A_ receptors (de Wit et al., 2001; Braden et al., 2010). These results may point to reasons why the combination of E2 and mPRO altered performance under cholinergic blockade compared to E2 alone.

The results of the present study reveal that mPRO produces a less pronounced reduction in cognitive performance compared to MPA. An explanation for the difference in the impact of mPRO and MPA on cognitive performance may be found in their pharmacological differences. MPA has been identified as having a much higher affinity with the progesterone receptor compared to progesterone, as well as having greater affinity with both the androgenic and glucocorticoid receptors (Kuhl, 2005; Stanczyk et al., 2013). This higher affinity may result in greater off-target activation, leading to potentially more interference, possibly through glutamatergic mechanisms (Nilsen et al., 2006). Additionally, while MPA does not bind to estrogen receptors, it has been identified as interfering with the impact of E2 to regulate brain mitochondrial function (Nilsen and Brinton, 2003; Irwin et al., 2011). Comparatively, mPRO has not been shown to disrupt downstream functions of E2 in the brain (Nilsen and Brinton, 2003). Taken together, these findings may indicate why in the present study we did not see a consistent decrement in performance when participants were administered E2 + mPRO.

The present study’s findings also have implications for how HT and the cholinergic system interact after menopause. As the use of progesterone/progestin in HT is part of the standard clinical care of postmenopausal women who have not had a hysterectomy, the understanding of the effects that mPRO has on cholinergically mediated cognitive performance is important for understanding whether long-term effects may arise from the use of mPRO in the wider population. While not comprehensively detrimental to performance, the fact that the combination of E2 and mPRO resulted in reduced performance under more effortful conditions is important. Given that the cholinergic system is modulated by E2, the interaction of mPRO and E2 is one that encourages further investigation. It would be important to know whether these effects are replicated across other progestins, and also whether the use of these progestins, including mPRO, may influence the risk of cognitive decline. Previously, we have found that postmenopausal women endorsing greater numbers of subjective cognitive complaints need to engage in greater cortical activation during effortful tasks (Dumas et al., 2013). Moreover, we have found that these women performed worse under cholinergic blockade, particularly on more effortful tasks, as they were unable to engage the cholinergic system to compensate for increased demands (Conley et al., 2022). Indeed, the endorsement of subjective cognitive complaints has been observed as a risk factor for the future development of AD (Jessen et al., 2010; Jessen, 2014). While beyond the scope of the current study, future research should investigate if women who report more cognitive changes after menopause are at an increased risk of future cognitive decline if they also take a progestin.

There are a number of limitations to the present study. Due to the nature of this intensive, within-subjects design, the sample size for the present study was small, which limits the generalizability of the results. In addition, due to the wide age range of participants, we are also unable to examine the use of combined E2 + mPRO during the first years following the menopause transition, which has been referred to as the “critical window” (Whitmer et al., 2011; Maki, 2013). Prior HT use is also a consideration, as the majority of participants had previously used HT, although to be eligible for the study they had to have been without HT for at least 1 year. Also, while we can postulate that MPA may be more detrimental, we cannot make predictions as to how a different progestin than mPRO, such as MPA, may have affected performance. Due to the multiple assessments of the same cognitive battery across the study period, we cannot completely rule out any practice or training effects on cognitive performance, however, the administration of cholinergic antagonists was consistently effective at blunting performance, so it does not seem that practice effects were able to overcome the effects of cholinergic blockade. There are also a number of strengths of the present study. The present study is a fully within-subjects design, with every participant completing both treatment phases and the subsequent challenge days. Every participant is therefore their direct comparator at each stage of the analysis. The study also uses a long washout period between testing phases to ensure a lack of carry-over effects from the first treatment phase. Due in part to these strengths, we believe that this work contributes to an understanding of the effects of the combination regimen of mPRO and E2 on the performance of cognitively unimpaired postmenopausal women in the presence of cholinergic blockade.

In conclusion, the present study showed that combining mPRO with E2 showed that under more effortful conditions of higher cholinergic blockade or increased task difficulty, mPRO was detrimental to the cognitive performance of cognitively unimpaired postmenopausal women, while not affecting other, less challenging tasks. While taking E2 + mPRO treatment and under cholinergic blockade, participants responded worse on the CFF task, more conservatively on the 3-back task, and slower on the Stroop task, compared to when they were taking E2 + PLC. We hypothesize that unlike the more consistent detriment of MPA on cognitive performance, the impact of mPRO on cholinergic compensation is determined by the requirements of a task, with greater required effort resulting in a ceiling of the ability to compensate. Future studies should examine whether different forms of progestin other than micronized progesterone have the same effect on cholinergically-mediated cognitive performance in postmenopausal women. Additionally, future studies should focus on underlying cholinergic integrity with structural and functional imaging methods to better characterize the deterioration of cholinergic tone in women following menopause, and how that may relate to future cognitive impairment.

## Data availability statement

The raw data supporting the conclusions of this article will be made available by the authors, without undue reservation.

## Ethics statement

The studies involving humans were approved by the University of Vermont Institutional Review Board. The studies were conducted in accordance with the local legislation and institutional requirements. The participants provided their written informed consent to participate in this study.

## Author contributions

AC: Writing – review & editing, Writing – original draft, Formal analysis, Data curation. JV: Writing – review & editing. JJ: Writing – review & editing, Investigation. JD: Writing – review & editing, Methodology, Investigation, Data curation, Conceptualization. PN: Writing – review & editing, Methodology, Investigation, Funding acquisition, Conceptualization.
