# Supplementary material for: Effect of estradiol with or without micronized progesterone on cholinergic-related cognitive performance in postmenopausal women
Source: Front Neurosci. 2024 Aug 8;18:1428675. doi: 10.3389/fnins.2024.1428675 (PMC11342399; doi:10.3389/fnins.2024.1428675)
Supplement: Supplementary file 1 [file Data_Sheet_1.docx]

Supplementary Material

# Specific criteria for exclusion:

Contraindications for hormone replacement and are history of breast cancer or E2-dependent neoplasia; blood pressure > 160/100 (untreated); history of deep vein thrombosis or other thromboembolic disease; hepatoma; severe migraines or stroke on oral contraceptives; current use of barbiturates, rifampin, insulin, carbamezepine, oral hypoglycemics, antidepressants, or lipid-lowering drugs; known intolerance to conjugated E2s; diabetes; untreated thyroid disease; clinical osteoporosis; severe menopausal symptoms. In addition, the following exclusions apply to the challenge drugs: heavy alcohol or coffee use, significant cardiovascular disease, asthma, active peptic ulcer, hyperthyroidism, pyloric stenosis, narrow-angle glaucoma, and epilepsy.

# Non-Challenge Day Cognitive Performance

| Task | Variable | Pre-Treatment Baseline | Post-First Treatment Re-Baseline |
| --- | --- | --- | --- |
| CFF | Median Ascending (Hz) | 28.2 (3.88) | 30.2 (3.77) |
|  | Median Descending | 32.5 (6.12) | 31.8 (4.81) |
| CRT | Median Total RT (ms) | 841 (94.3) | 854 (148) |
|  | Median Recognition RT | 427 (41.4) | 434 (43.9) |
|  | Median Motor RT | 398 (68.5) | 415 (111) |
| N-back | 0-back Sensitivity (d’) | 5.65 (0.93) | 4.53 (3.68) |
|  | 1-back Sensitivity | 4.38 (1.23) | 3.63 (3.78) |
|  | 2-back Sensitivity | 2.86 (1.1) | 2.18 (3.05) |
|  | 3-back Sensitivity | 2.22 (0.78) | 1.65 (1.68) |
|  | 0-back Bias (C) | 0.2 (0.5) | 0.06 (0.45) |
|  | 1-back Bias | 0.38 (0.46) | -0.01 (0.55) |
|  | 2-back Bias | -0.09 (0.47) | 0.1 (0.63) |
|  | 3-back Bias | 0.41 (0.36) | 0.47 (0.55) |
| Recognition Memory | Sensitivity (d’) | 2.77 (0.91) | 2.7 (0.82) |
|  | Bias (C) | 0.26 (0.63) | 0.28 (0.51) |
| Stroop Task | Congruent Accuracy (% correct) | 95.1 (1.05) | 95.6 (0.82) |
|  | Incongruent Accuracy | 97.2 (4.07) | 98.1 (1.58) |
|  | Congruent RT (ms) | 692 (125) | 686(128) |
|  | Incongruent RT | 1116 (331) | 997 (296) |

Notes. CFF, critical flicker fusion task; CRT, choice reaction time task. Numbers represent mean scores on the placebo challenge day with standard deviations in parentheses.
